# Efficiency of laparoscopic artificial insemination in goats: impact of laparoscopic insemination sheath needle design

**DOI:** 10.3389/fvets.2025.1579540

**Published:** 2025-06-27

**Authors:** Ting-Chieh Kang, Hsin-Hung Lin, Hsiu-Lien Lin, Pei-Chun Tsai, Yu-Hsin Chen, Fung-Hsiang Chu, Kai-Fei Tseng, I-Ling Lai, Perng-Chih Shen

**Affiliations:** ^1^Taiwan Livestock Research Institute, Ministry of Agriculture, Tainan, Taiwan; ^2^Graduate Institute of Bioresources, National Pingtung University of Science and Technology, Pingtung, Taiwan; ^3^Department of Animal Science, National Pingtung University of Science and Technology, Neipu, Ping-tung, Taiwan

**Keywords:** goat, laparoscopic artificial insemination, sheath needle design, pregnancy rate, kidding rate, average litter size

## Abstract

**Introduction:**

Laparoscopic artificial insemination (LAI) is widely used in goat breeding, yet the influence of sheath needle design on reproductive outcomes remains unclear. This study aimed to evaluate the effects of three different LAI sheath designs (IMV, Minitube, and TLRI) on pregnancy rate, kidding rate, and average litter size in Alpine does.

**Methods:**

A total of 300 Alpine does (2–3 years old) were enrolled in two experiments. In Experiment 1, does were inseminated into either the middle-horn (M-H) or the horn-body junction (H-BJ) of one uterine horn using each of the three sheath types. In Experiment 2, the IMV and TLRI sheaths were used to deposit semen either unilaterally or bilaterally. Pregnancy rate, kidding rate, and average litter size were recorded for all treatment groups. Statistical significance was set at *p* < 0.05.

**Results:**

In Experiment 1, overall pregnancy rates with TLRI and IMV sheaths were significantly higher than with the Minitube sheath (*p* < 0.05) regardless of site. The lowest kidding rate occurred with the Minitube sheath at the H-BJ site (*p* < 0.05), while the highest was observed with the TLRI sheath at the M-H site (*p* < 0.05). No significant differences in kidding rate or average litter size were found among the remaining groups (*p* > 0.05). In Experiment 2, there were no significant differences in pregnancy rate, kidding rate, or average litter size between unilateral and bilateral inseminations using either the IMV or TLRI sheath (*p* > 0.05).

**Discussion:**

These findings demonstrate that both TLRI and IMV sheath designs outperform the Minitube sheath in terms of pregnancy and kidding rates, particularly when targeting the M-H site. Moreover, unilateral insemination with these sheaths does not compromise reproductive performance and offers a time-efficient alternative to bilateral deposition. Adoption of optimal sheath designs and insemination strategies can enhance LAI efficiency and success in goat breeding programs.

## 1 Introduction

Artificial insemination (AI) is the most convenient assisted reproductive technology (ART) for promoting genetic improvement in goat herds. However, in practice, success rates often remain between 55% and 59.5% (1). The goat cervix is a highly complex fibrous structure with 4 to 7 folds, creating a narrow lumen. Typically, the second fold is more asymmetrical than the first and third folds, leading to a narrowing of the cervical canal (2–5). This anatomical structure obstructs the possibility of inserting the catheter deeply into the uterine horn or uterine body during AI (6). Forced entry can easily injure the cervical wall (7, 8), which can trigger an immune defense response, leading to pregnancy termination between days 3 and 14 (9). To overcome the limitations, several improvements have been proposed in the design of conventional transcervical AI equipment. Falchi et al. (10) conducted experiments focusing on the curvature angle at the distal end of the insemination catheter and found that a 5.0 mm bend yielded the most favorable outcome for facilitating rapid and deep intrauterine semen deposition. Their results indicated that, in most cases, semen was successfully deposited within the uterine body rather than remaining in the cervical canal. Additionally, Alvarez et al. (3) introduced an unidirectional check valve at the proximal end of the catheter. This structural modification effectively prevented semen reflux and promoted deeper penetration into the cervical canal, without necessitating alterations to the standard intra-cervical insemination protocol. Collectively, these innovations have been shown to significantly enhance pregnancy rates and increase the number of offspring per insemination.

Many studies have found that placing sperm in a deeper location during AI significantly improves pregnancy rates in ewes (11–13). However, due to the natural obstruction of the goat cervix, placing sperm deeper, such as in the uterine horn or uterine body, is highly challenging.

LAI is an advanced assisted reproductive technology particularly suited for small ruminants such as sheep and goats. This technique, assisted by specialized instruments, directly deposits sperm at the uterine horn or uterine body, bypassing the physical barriers of the cervix. It also reduces the distance and time required for sperm to reach the oviduct, thereby significantly increasing pregnancy rates (14, 15). LAI can also achieve a pregnancy rate of 60–80% using lower sperm concentrations (5 × 10^6^ spermatozoa/mL) compared to cervical AI (55–59.5%) (6, 16). In addition to greatly improving sperm utilization efficiency, this technology makes it feasible to use sex-sorted semen for AI (17, 18). However, LAI is not without its drawbacks, including high equipment costs, technical expertise requirements for operators, and greater manpower demands, all of which are bottlenecks to its application in economic animals and remain challenges to be addressed.

Many factors influence the success rate of LAI, including regional differences, breeding season, individual rams, ejaculate sequence of the same ram, depth of cervical penetration during insemination (3), estrus synchronization protocols (19, 20), sperm quality (21–24), operator experience (24, 25), age, breed, and management practices (6). Few studies, however, have focused on the needle design of laparoscopic insemination sheaths.

Recent modifications to LAI cannulas have improved insufflation control and semen-deposit precision. Standard 5 mm trocar–cannula sets now employ external insemination guns within sheaths to minimize leakage and target uterine horns more effectively (20). Insights from transcervical catheter tip geometry, using bent tips (3.5, 5.0, 8.0 mm), suggest similar benefits for laparoscopic applications (10). Additionally, hydrodynamic double-lumen needle designs inform internal flow optimization for improved semen distribution (26). Combined with consistent training and equipment standardization, these innovations enhance procedural reproducibility and pregnancy outcomes (27). In human medicine, the geometry of needles (e.g., number of bevels at the needle tip) significantly affects the forces and energy required for insertion and withdrawal. Needles with five bevels require significantly less penetration and drag force compared to those with three bevels, potentially reducing pain experienced by patients (28). Smaller-diameter and shorter needles significantly reduce the pressure needed for injection, thereby reducing the physical burden on operators and pain for patients (29). Short and fine needles (e.g., 32G × 4 mm) used in insulin injections are better tolerated by patients, enhance drug absorption, and cause less pain (28). Currently, the commercial laparoscopic insemination sheaths available are primarily produced by IMV (France) and Minitube (Germany), with notable differences in design. The IMV sheath, marketed as Aspic for mini straw (Ref. 005546), is used with the Ovine transcap with guide (Ref. 007188) and allows frozen semen straws to be directly mounted after thawing. Minitube’s product, the Robertson standard pipette (Ref. 23700/2200), is used with the Lap AI gun for Robertson pipettes (Ref. 23700/2205) or directly connected to a syringe at the rear end. A new product developed by the Taiwan Livestock Research Institute (TLRI) is compatible with both 0.25 mL and 0.5 mL frozen semen straws and can be used with any brand of universal AI gun for sheep.

However, there is limited discussion on the specific parameters of needle tips in LAI sheaths. Therefore, this study aims to compare the needle design parameters of three types of LAI equipment and evaluate their efficiency in terms of pregnancy rate, lambing rate, and average litter size following LAI at different positions in the uterine horn and unilateral versus bilateral insemination in goats.

## 2 Materials and methods

### 2.1 Animals

All experimental animals used in this study were sourced exclusively from our own research farm, and no animals were obtained from other owners. Prior to the start of the experiment, we applied for the use of experimental animals, which is reviewed and approved by our Experimental Animal Care and Use Committee. An approval letter is then issued accordingly. This study included 300 Alpine does aged 2–3 years, all with at least one parity. Trial 1 utilized 180 does, while Trial 2 included 120 does. All animals were screened using a five-point body condition scoring system, selecting those with scores between 2.5 and 3. Does with a history of abortion or failed breeding during the previous cycle were excluded. Animal use was approved by the Institutional Animal Care and Use Committees (IACUC) under authorization numbers N°-HUA-IACUC-101-07 and N°-LRI-IACUC-102-003.

### 2.2 Experimental design

#### 2.2.1 Experiment 1

Three LAI sheaths were evaluated at two uterine horn positions middle of uterine horn (M-H) and the uterine horn-body junction (H-BJ)—based on pregnancy rate, kidding rate, and average litter size.

#### 2.2.2 Experiment 2

The two best-performing sheaths from Experiment 1 were used to compare unilateral and bilateral uterine horn LAI. Pregnancy rate, kidding rate, and average litter size were measured 45 days post-insemination. For bilateral insemination, the semen concentration and volume matched those used in unilateral insemination.

### 2.3 Semen preparation and semen diluent

Cryopreserved semen from a single Alpine buck was sourced from the Hengchun Branch of the Livestock Research Institute. Semen was stored in liquid nitrogen and thawed in a 37°C water bath for 30 s before use. Post-thaw quality was assessed with a computer-assisted sperm analyzer (Sperm Class Analyzer® CASA System, MICROPTIC, Barcelona, Spain). Only semen with ≥50% motility and ≥50% progressive motility was used. The final semen concentration was adjusted to 50 × 10^6^ cells/0.25 mL for all trials. The semen diluent was prepared using the following components per 100 mL: 2.42 g Tris (hydroxymethyl aminomethane) (T1503), 1.48 g citric acid (C0759), 1.00 g glucose (G8270), 1 mL penicillin–streptomycin solution (P4333), and 6% (dry matter basis) low-density lipoprotein (LDL). The final concentration of glycerol (G5516) in the diluent was adjusted to 7% (v/v). All chemicals used for semen extender preparation were obtained from Sigma-Aldrich.

### 2.4 Estrus synchronization

Estrus synchronization was performed using CIDR® (Controlled Internal Drug Release, EAZI-breed, Rydalmere, Australia), PMSG (Pregnant Mare’s Serum Gonadotropin, Prospec-Tany, Israel), and PGF2α (Estrumate, Vet Pharma Friesoythe Gmbh). On Day 0, CIDR® was inserted, followed by intramuscular injections of PGF2α (5.3 mg/0.5 mL) and PMSG (400 IU) on Day 9. CIDR® was removed on Day 11, and LAI was performed 55 h later. The dosage of PMSG was based on the studies by Tripan et al. (30).

### 2.5 Measurement and determination criteria for each parameter

*Pregnancy rate (%)*: At 45 days after laparoscopic artificial insemination, pregnancy was diagnosed using transrectal ultrasonography. An ultrasound scanner (Aloka, SSD-500, Japan) equipped with a transrectal probe (Aloka, linear type, 3.5 MHz, Japan) was used to detect uterine horn structures and fetal images for confirmation of pregnancy.
*Kidding rate (%)*: Calculated based on does that gave birth within 150 ± 7 days after laparoscopic artificial insemination.
*Average litter size*: Number of kids born divided by the number of does that gave birth.

### 2.6 Laparoscopic equipment

The laparoscopic setup included a rigid laparoscope (6 mm external diameter, 0° viewing angle), a cold light source, a video imaging system, and a CO₂ insufflator (Karl Storz, Germany). Two 6 mm trocars were used for laparoscope and insemination needle placement.

### 2.7 Anesthesia

Does were fasted for 24 h before surgery. Preoperative muscle relaxation was induced with xylazine (0.2 mg/kg, Rompun®, Bayer, Germany) and atropine sulfate (0.025 mg/kg, Xindong Biotech, Taiwan). Sedation was administered intravenously using Zoletil 50® (0.75 mg/kg, Virbac, France), followed by maintenance anesthesia with 2–3% isoflurane (Piramal Healthcare, India) in oxygen (2–4 L/min). Blood oxygen levels and respiratory rates were monitored during the procedure. Anesthesia lasted 10–15 min, and recovery to standing occurred within 30–60 min.

### 2.8 Surgical positioning

Does were positioned in the Trendelenburg position (head-down, 60° incline) on a surgical table, with hind legs secured for support.

### 2.9 Laparoscopic procedure and operator

The surgical area was shaved, sterilized with povidone-iodine and 75% alcohol, and locally anesthetized with 2–3 mL of 20 mg/mL lidocaine. Two incisions (~1 cm) were made ~2–3 cm anterior to the nipples, and CO₂ was used to inflate the abdominal cavity. The laparoscope was inserted through one cannula and the insemination sheath through the other. Wounds were closed with nonabsorbable silk sutures (Silkam, B. Braun, Spain) and treated with intramuscular injections of Penisol (0.2 mL/kg, China Chemical & Pharmaceutical Co., Ltd., Taiwan) once daily for 5 days to prevent infection. In order to minimize variability due to operator differences, all laparoscopic artificial insemination procedures were conducted by the same technician throughout the study.

### 2.10 LAI sheaths

Three LAI sheaths were tested.

#### 2.10.1 IMV

Aspic for Mini Straw (Ref. 005546) with Ovine Transcap with Guide (Ref. 007188); needle length: 60 mm; internal diameter: 0.5 mm; bevel angle: 23° (Supplementary Figure 1B).

#### 2.10.2 Minitube

Robertson Standard Pipette (Ref. 23700/2200) with Lap AI Gun (Ref. 23700/2205); needle length: 45 mm; internal diameter: 0.5 mm; bevel angle: 35° (Supplementary Figure 1A).

#### 2.10.3 TLRI

Developed by the Taiwan Livestock Research Institute; compatible with 0.25 mL and 0.5 mL semen straws; needle length: 55 mm; internal diameter: 0.5 mm; bevel angle: 30°. The laparoscopic insemination sheath used in this study was a prototype developed by the Taiwan Livestock Research Institute (TLRI). This device is still at the laboratory development stage and is not yet commercially available (supplementary Figures 1C, 2).

Semen was prepared at a concentration of 50 × 10^6^ spermatozoa/0.25 mL, refilled into 0.25 mL straws, and thawed before use.

### 2.11 Statistical analysis

Statistical analyses were conducted using SAS Enterprise Guide 7.1 (SAS Institute Inc., Cary, NC, United States). For the three continuous variables (Pregnant rate, Kidding rate, and Average litter size), comparison among different treatments were done by one-way analysis of variance (ANOVA), followed by Tukey’s HSD (Honestly Significant Difference) test for multiple comparisons of pregnancy rate, kidding rate, and average litter size. Statistical significance was set at *p* < 0.05.

## 3 Results

### 3.1 Experiment 1: comparison of three LAI sheath at different uterine horn sites

#### 3.1.1 Pregnancy rate

The pregnancy rates for the TLRI and IMV sheath were significantly higher than those of the Minitube sheath at both uterine horn sites. At mid-horn and horn-body junction, the pregnancy rates for TLRI and IMV were 62.67 ± 1.45 [95% CI: 59.82–65.51] vs. 63.00 ± 0.58 [95% CI: 61.87–64.13] and 62.33 ± 0.88 [95% CI: 60.60–64.06] vs. 62.67 ± 0.33 [95% CI: 62.01–63.32], respectively, with no significant difference between them but significantly higher than Minitube (54.93 ± 0.78 [95% CI: 53.40–56.46] vs. 52.86 ± 1.00 [95% CI: 50.89–54.83]). These findings suggest that the designs of the TLRI and IMV sheath are better suited for the uterine horn structure in goats, enhancing sperm deposition efficiency and improving fertilization success rates. In contrast, the lower pregnancy rate for the Minitube sheath may be attributed to its larger needle diameter and greater tip angle (Table 1).

**Table 1:** Efficiency of insemination brand on pregnancy rate, kidding rate, and average litter size.

Brand	Position	Number	Pregnancy rate, % (95% CI, %)	Kidding rate (%) (95% CI, %)	Average litter size (95% CI)
TLRI	M-H	30	62.67 ± 1.45^a^ (59.82–65.51)	86.57 ± 1.73^ab^ (83.17–89.96)	2.05 ± 0.04 (1.98–2.13)
	H-BJ	30	63.00 ± 0.58^a^ (61.87–64.13)	87.30 ± 1.04^a^ (85.25–89.35)	2.10 ± 0.06 (1.99–2.21)
IMV	M-H	30	62.33 ± 0.88^a^ (60.60–64.06)	85.40 ± 0.57^ab^ (84.29–86.51)	2.10 ± 0.02 (2.07–2.14)
	H-BJ	30	62.67 ± 0.33^a^ (62.01–63.32)	86.40 ± 0.47^ab^ (85.47–87.33)	2.13 ± 0.12 (1.90–2.37)
Minitube	M-H	30	54.93 ± 0.78^b^ (53.40–56.46)	80.20 ± 1.29^c^ (77.68–82.72)	2.07 ± 0.07 (1.93–2.21)
	H-BJ	30	52.86 ± 1.00^b^ (50.89–54.83)	81.62 ± 0.61^bc^ (80.43–82.81)	2.05 ± 0.05 (1.96–2.14)

M-H, middle of the uterine horn; H-BJ, uterine horn–body junction. Values are presented as the mean ± standard error. ^abc^Values with different lowercase superscripts in the same column indicate significant differences (*p* < 0.05).

#### 3.1.2 Kidding rate

The kidding rates for each group are presented in Table 1. The lowest rate was observed with the Minitube sheath at the M-H position (80.20 ± 1.29 [95% CI: 77.68–82.72]), which was significantly lower than that of the TLRI sheath at the mid-horn (87.30 ± 1.04% [95% CI: 85.25–89.35]; *p* < 0.05). The kidding rates for the TLRI sheath at the H-BJ (86.57 ± 1.73% [95% CI: 83.17–89.96]), the IMV sheath at both of the M-H (85.40 ± 0.57% [95% CI: 84.29–86.51]) and H-BJ (86.40 ± 0.47% [95% CI: 85.47–87.33]), the Minitube sheath at the H-BJ (81.62 ± 0.61% [95% CI: 80.43–82.81]), and the ranged from 81.62 to 86.57%, with no significant differences (*p* > 0.05). The values of kidding rates for each treatment group fell within the 95% confidence intervals (Table 1).

#### 3.1.3 Average litter size

Variations in average litter size across different sheath and insemination sites were minimal, ranging from 2.05 to 2.13 kids (Table 1). This suggests that the choice of insemination device has no significant impact on average litter size. The values of average litter size for each treatment group fell within the 95% confidence intervals.

### 3.2 Experiment 2: evaluation of unilateral vs. bilateral uterine horn insemination

Based on the results of Experiment 1, the two best-performing sheaths (IMV and TLRI) were used to evaluate pregnancy rates, kidding rates, and average litter sizes following unilateral or bilateral uterine horn insemination (Table 2).

**Table 2:** The effect of unilateral vs. bilateral uterine horn insemination on pregnancy rate, kidding rate, and average litter size.

Position	Brand	Number	Pregnancy rate, % (95% CI, %)	Kidding rate, % (95% CI, %)	Average litter size (95% CI, %)
Unilateral uterine horn	IMV	30	63.67 ± 2.96 (50.92–76.41)	89.00 ± 0.58 (86.52–91.48)	2.03 ± 0.09 (1.65–2.41)
	TLRI	30	64.33 ± 1.20 (59.16–69.50)	88.33 ± 1.67 (81.16–95.50)	1.97 ± 0.09 (1.59–2.35)
Bilateral uterine horn	IMV	30	64.00 ± 0.58 61.52–66.48	90.33 ± 0.88 (86.54–94.13)	2.10 ± 0.06 (1.85–2.35)
	TLRI	30	63.67 ± 0.88 (59.87–67.46)	90.00 ± 0.58 (87.52–92.48)	2.07 ± 0.07 (1.78–2.35)

Values are presented as the mean ± standard error.

#### 3.2.1 Pregnancy rate

The pregnancy rates for unilateral insemination were 63.67% ± 2.96 [95% CI: 50.92–76.41] (IMV) and 64.33% ± 1.20 [95% CI: 59.16–69.50] (TLRI). For bilateral insemination, the rates were 64.00% ± 0.58 [95% CI: 61.52–66.48] (IMV) and 63.67% ± 0.88 [95% CI: 59.87–67.46] (TLRI). There were no significant differences between unilateral and bilateral insemination. Although bilateral insemination theoretically provides more fertilization opportunities, the results indicate that unilateral insemination is sufficient for achieving satisfactory pregnancy rates. This may be related to the distribution characteristics of semen within the goat uterus, where the concentration and distribution of semen in unilateral insemination meet fertilization requirements.

#### 3.2.2 Kidding rate

The kidding rates for unilateral insemination, the rates were 89.00% ± 0.58 [95% CI: 86.52–91.48] (IMV) and 88.33% ± 1.67 [95% CI: 81.16–95.50] (TLRI). For bilateral insemination were 90.33% ± 0.88 [95% CI: 86.54–94.13] (IMV) and 90.00% ± 0.58 [95% CI: 87.52–92.48] (TLRI). No significant differences were observed between the groups.

#### 3.2.3 Average litter size

The average litter sizes for bilateral insemination were 2.10 ± 0.06 (IMV) and 2.07 ± 0.07 (TLRI). For unilateral insemination, the sizes were 2.03 ± 0.09 (IMV) and 1.97 ± 0.09 (TLRI). No significant differences were observed.

Statistical analysis showed no significant differences among the values in each treatment group.

## 4 Discussion

### 4.1 Comparison of different insemination position within a single uterine horn

In this experiment, semen (0.25 mL) containing 50 million sperm was injected into two different locations: the mid-section of a unilateral uterine horn and the junction between the uterine horn and uterine body. To compare the performance of different laparoscopic artificial insemination (LAI) devices, the pregnancy rate was evaluated on 45 days post-insemination, the kidding rate after conception, and the average number of fetuses. The results showed no significant difference in pregnancy rates between the M-H and H-BJ positions when using the Minitube insemination pipette, and the same phenomenon was observed with IMV and TLRI devices. However, Minitube’s overall performance in terms of pregnancy rate was significantly lower than that of IMV and TLRI (Table 1). Regarding the LAI site, this outcome aligns with the findings of Andersson et al. (31), who noted no significant difference in conception rates between insemination in the uterine body (46.4%) and the uterine horn (43.3%) in dairy cows. However, this contrasts with the findings of Grave et al. (32), who observed a higher success rate for insemination in the uterine body (62.9%) compared to bilateral uterine horn insemination (54.2%).

As for the kidding rate presented in Table 1, the lowest rate was observed with the Minitube sheath at the M-H position (80.20 ± 1.29), which was significantly lower than that of the TLRI sheath at the M-H (87.30 ± 1.04%). The kidding rates for the TLRI sheath at the H-BJ (86.57 ± 1.73%), the IMV sheath at both of the M-H (85.40 ± 0.57%) and H-BJ (86.40 ± 0.47%), the Minitube sheath at the H-BJ (81.62 ± 0.61%), and the ranged from 81.62 to 86.57%, with no significant differences. Existing research on LAI has generally found that most performance differences lie in pregnancy outcomes, with no significant variation in kidding rates (20, 33).Generally, LAI techniques demand a high level of skills, and operator experiences, which directly impacts the success rate. Previous studies have confirmed that experienced practitioners achieve significantly higher success rates (32). Skilled operators are able to minimize complications such as abdominal puncture and internal bleeding, while also completing more procedures within the same timeframe, thereby improving overall efficiency (20). This study also further analyzed the design parameters of the various device needles, including needle length, tip angle, the pressure required for semen injection, ease of penetration, and depth control capability. The findings underscored the critical importance of operator’s proficiency and equipment optimization in achieving stable and successful reproductive outcomes in goats.

### 4.2 Unilateral vs. bilateral uterine horn insemination

This study utilized the IMV and TLRI sheaths, which demonstrated superior pregnancy rates in Experiment 1, to evaluate the outcomes of unilateral or bilateral mid-horn insemination. Results showed no significant differences in pregnancy rate, kidding rate, or average litter size between the two methods 45 days post-insemination (Table 2). These findings contrast with those reported by Evans and Maxwell (15), who observed higher pregnancy rates in ewes following bilateral uterine horn insemination via laparoscopic artificial insemination (LAI). However, a study on synchronized Merino ewes investigated the effect of unilateral versus bilateral uterine horn insemination using frozen–thawed semen administered through LAI. A total of 217 ewes were divided into unilateral (*n* = 107) and bilateral (*n* = 110) groups. Pregnancy rates were 52.3 and 55.5%, respectively, with no significant differences between the groups (34). A similar outcome was reported in goats (35), aligning with the present study’s results. In summary, all three LAI sheaths evaluated in this study successfully facilitated laparoscopic insemination in goats, with the IMV and TLRI sheaths providing higher efficiency due to optimized needle designs. The injection site within the uterine horn and the use of unilateral versus bilateral insemination had no significant impact on pregnancy outcomes. The critical factor in successful LAI lies in accurately depositing semen into the uterine cavity. Unilateral mid-horn insemination offers a more time-efficient method for LAI in goats without compromising reproductive success.

### 4.3 Influence of needle design on LAI success rates

#### 4.3.1 Needle inner diameter (𝐷)

According to Poiseuille’s law, flow rate (𝑄) is proportional to the fourth power of the needle’s inner diameter:


$$Q=\frac{\mathrm{\pi \Delta P}D^{4}}{128\mathrm{\mu L}}$$

Where ∆P, Pressure difference; *μ*, Fluid viscosity; L, Needle length.

An increase in needle diameter significantly improves flow rate at a given pressure. However, in this study, all sheath had the same inner diameter (0.5 mm), making flow rate differences dependent on needle length and tip angle.

#### 4.3.2 Needle length (𝐿)

Flow rate is inversely proportional to needle length:


$$Q\propto \frac{1}{L}$$

Longer needles reduce flow rate due to increased resistance, requiring higher pressures to achieve similar flow rates. Among the sheath tested, Minitube had the shortest needle length, followed by TLRI, with IMV having the longest.

#### 4.3.3 Needle tip angle (𝜃)

Larger tip angles (flatter) reduce resistance to fluid flow, making it easier for fluids to enter target tissues. Smaller tip angles (sharper) reduce insertion resistance but may limit flow rate. In this study, IMV had the smallest tip angle (23°), TLRI was intermediate (30°), and Minitube had the largest (35°).

Using Poiseuille’s law, the pressure required (ΔP) to maintain a given flow rate can be expressed as:


$$\mathrm{\Delta P}=\frac{8\mathrm{\mu LQ}}{\pi r^{4}}$$

Where r is the inner radius (0.25 mm for all needles).

In this study, the inner diameter and flow rate of LAI sheaths from the three examined brands were identical. Consequently, the primary factors determining pressure requirements were needle length (𝐿) and needle tip bevel angle, as the latter influences fluid entry and exit efficiency. The bevel angle affects the resistance encountered by fluid passing through the needle: a smaller angle (IMV, 23°) results in a longer bevel, increasing fluid outlet resistance and subsequently requiring greater pressure. In contrast, a larger angle (Minitube, 35°) shortens the bevel, reducing fluid outlet resistance and thereby lowering pressure demands. The intermediate angle (TLRI, 30°) falls between these two extremes, resulting in moderate outlet resistance. The diameter and length of the needle have been demonstrated to directly influence pain perception. Furthermore, additional factors, including needle tip shape, sharpness, insertion angle, gliding performance, and the number of friction points (i.e., the number of bevels), are critical in determining both the needle’s ability to penetrate tissue and its impact on pain sensation (36). Beyond needle length, inner diameter, and bevel angle, the number of bevels also plays a crucial role in penetration efficiency and pain levels. A comparative study on insulin pen needles used by diabetic patients found that modified asymmetric three-bevel needles outperformed traditional three-bevel needles in terms of both force requirements and pain perception (37). Additionally, research on bevel quantity has shown that five-bevel needles exhibit superior penetration force and lower withdrawal resistance compared to three-bevel needles. Experimental assessments of the insertion-withdrawal cycle indicated that five-bevel needles require 11.5 to 29% less energy than three-bevel needles, suggesting that less energy is transferred to the surrounding tissue. Clinically, this implies that five-bevel needles effectively reduce the force and energy associated with needle friction, thereby minimizing patient discomfort during injection (28, 38). These design improvements not only enhance surgical efficiency but also contribute significantly to animal welfare. Based on the derived formulas, the parameters and operational characteristics of laparoscopic artificial insemination sheaths from different manufacturers are summarized in Table 3.

**Table 3:** Needle and practical operation characteristics of different brands.

Parameter	IMV	TLRI	Minitube
Needle inner diameter (mm)	0.5	0.5	0.5
Needle length (mm)	6	5.5	4.5
Needle bevel angle (°)	23	30	35
Pressure requirement	Highest	Moderate	Lowest
Uterine horn wall penetrability	Good positioning, easy penetration	Moderate, suitable for smooth uterine walls	Poor, higher risk of blockage
Semen injection accuracy	Accurate	Accurate	Accurate
Risk of blockage after penetration	Low probability of complete blockage due to small bevel angle	Moderate risk of blockage	High risk of blockage

During the procedure, the successful deposition of semen into the uterine horn cavity can be confirmed by observing whether the uterine horn exhibits expansion. Based on the above results, we compared key parameters of the insemination needle, including inner diameter, length, and bevel angle. Since the inner diameters were identical across brands, we found that a longer needle and a smaller bevel angle enhanced the efficiency of LAI.

In conclusion, all three laparoscopic artificial insemination sheaths evaluated in this study were successfully applied in LAI in goats, with the IMV and TLRI sheaths demonstrating superior performance. The pregnancy rate was not significantly affected by whether semen was deposited unilaterally or bilaterally in the uterine horn. Precise semen deposition in the uterine horn cavity is critical, and unilateral insemination can reduce the procedure time for LAI. Additionally, an appropriately designed needle can significantly enhance the efficiency of laparoscopic artificial insemination.

## Data Availability

All underlying data supporting the results of this study have been deposited in the Zenodo repository, available at https://zenodo.org/records/15654973.

## References

[1] Richardson LL Hanrahan JP Donovan A Martí JI Fair S Evans ACO. Effect of site of deposition on the fertility of sheep inseminated with frozen-thawed semen. Anim Reprod Sci. (2012) 131:160–4. doi: 10.1016/j.anireprosci.2012.03.006, PMID: 22483943

[2] Fukui Y Roberts EM. Further studies on non-surgical intrauterine technique for artificial insemination in the ewe. Theriogenology. (1978) 10:381–93. doi: 10.1016/0093-691X(78)90042-0

[3] Álvarez M Chamorro CA Kaabi M Anel-López L Boixo JC Anel E. Design and in vivo evaluation of two adapted catheters for intrauterine transcervical insemination in sheep. Theriogenology. (2012) 77:1232–40. doi: 10.1016/j.anireprosci.2012.03.001 22483334

[4] Naqvi SMK Pandey GK Gautam KK Joshi A Geethalakshmi V Mittal JP. Evaluation of gross anatomical features of cervix of tropical sheep using cervical silicone moulds. Anim Reprod Sci. (2005) 85:337–44. doi: 10.1016/j.anireprosci.2003.10.007 15581516

[5] Kaabi M Álvarez M Anel E Chamorro CA Boixo JC de Paz P. Influence of breed and age on morphometry and depth of inseminating catheter penetration in the ewe cervix: a postmortem study. Theriogenology. (2006) 66:1876–83. doi: 10.1016/j.theriogenology.2006.04.039 16790269

[6] Anel L Kaabi M Abroug B Álvarez M Anel E Boixo JC. Factors influencing the success of vaginal and laparoscopic artificial insemination in churra ewes: a field assay. Theriogenology. (2005) 63:1235–47. doi: 10.1016/j.theriogenology.2004.07.001 15710206

[7] Campbell JW Harvey TG McDonald MF Sparksman RI. Transcervical insemination in sheep: an anatomical and histological evaluation. Theriogenology. (1996) 45:1535–44. doi: 10.1016/0093-691X(96)00121-5

[8] Wulster-Radcliffe MC Lewis GS. Development of a new transcervical artificial insemination method for sheep: effects of a new transcervical artificial insemination catheter and traversing the cervix on semen quality and fertility. Theriogenology. (2002) 58:1361–71. doi: 10.1016/S0093-691X(02)01042-7 12387349

[9] Wulster-Radcliffe MC Wang S Lewis GS. Transcervical artificial insemination in sheep: effects of a new transcervical artificial insemination instrument and traversing the cervix on pregnancy and lambing rates. Theriogenology. (2004) 62:990–1002. doi: 10.1016/j.theriogenology.2003.12.031 15289042

[10] Falchi L Zedda MT Pau S Ledda M Melosu V Rassu SPG. The design of a new catheter for transcervical artificial insemination in ewes. Animals. (2021) 11:3348. doi: 10.3390/ani11123348 34944124 PMC8698084

[11] Eppleston J Salamon S Moore NW Evans G. The depth of cervical insemination and site of intrauterine insemination and their relationship to the fertility of frozen-thawed ram semen. Anim Reprod Sci. (1994) 36:211–25.

[12] Halbert GW Dobson H Walton JS Sharpe P Buckrell BC. Field evaluation of a technique for transcervical intrauterine insemination of ewes. Theriogenology. (1990) 33:1231–43. doi: 10.1016/0093-691X(90)90041-Q 16726795

[13] Salamon S Maxwell WMC. Frozen storage of ram semen. Part II. Causes of low fertility after cervical insemination and methods of improvement. Anim Reprod Sci. (1995) 38:1–36. doi: 10.1016/0378-4320(94)01328-J

[14] Dun R. The cervix of the ewe. Its importance in artificial insemination of sheep. Aust Vet J. (1955) 31:101–3. doi: 10.1111/j.1751-0813.1955.tb05513.x

[15] Evans G. Maxwell W. M. Salamon’s artificial insemination of sheep and goats. Sydney:Butterworths (1987). 154–159.

[16] Fantinati P Zannoni A Bernardini C Webster N. Laparoscopic insemination technique with low numbers of spermatozoa in superovulated prepuberal gilts for biotechnological application. Theriogenology. (2005) 63:806–17. doi: 10.1016/j.theriogenology.2004.05.005, PMID: 15629799

[17] del Olmo D Parrilla I Sanchez-Osorio J Gomis J Angel MA Tarantini T. Successful laparoscopic insemination with a very low number of flow cytometrically sorted boar sperm in field conditions. Theriogenology. (2014) 81:315–20. doi: 10.1016/j.theriogenology.2013.09.031 24157229

[18] Sugai N Shipley C Rothrock-Gensler L Ellerbrock R Stewart J. Laparoscopic artificial insemination of white-tailed deer with fresh sex-sorted semen. Clin Theriogenol. (2024) 16:513. doi: 10.58292/CT.v16.10513

[19] Candappa IBR Bartlewski PM. A review of advances in artificial insemination (AI) and embryo transfer (ET) in sheep, with special reference to hormonal induction of cervical dilation and its implications for controlled animal reproduction and surgical techniques. Open Reprod Sci J. (2011) 3:162–75. doi: 10.2174/1874255601103010162

[20] Sathe SR. Laparoscopic artificial insemination technique in small ruminants—a procedure review. Front Vet Sci. (2018) 5:266. doi: 10.3389/fvets.2018.00266, PMID: 30406122 PMC6206429

[21] Faigl V Vass N Jávor A Kulcsár M Solti L Amiridis G. Artificial insemination of small ruminants—a review. Acta Vet Hung. (2012) 60:115–29. doi: 10.1556/avet.2012.010, PMID: 22366137

[22] Khan MT Ahmad E Yousaf MR Oneeb M Akhtar MS Irfan-ur-Rehman M. Optimising laparoscopic artificial insemination in lohi sheep: effects of timing, sperm concentration and body condition on fertility outcome in subtropical climates. Reprod Domest Anim. (2024) 59:e14725. doi: 10.1111/rda.14725 39315464

[23] McCappin M Murray RD. Some factors affecting pregnancy rate in ewes following laparoscopic artificial insemination. Vet Rec. (2011) 168:99. doi: 10.1136/vr.c5979 21493468

[24] Spanne EA de Graaf SP Rickard JP. A multivariate model for the prediction of pregnancy following laparoscopic artificial insemination of sheep. Nat Commun. (2024) 14:27556. doi: 10.1038/s41598-024-79253-x PMC11555048 39528692

[25] Karaka O Cemal I Yilmaz O Yilmaz M. Effect of laparoscopic insemination on reproductive performance of indigenous cine Capari sheep. Indian J Anim Sci. (2012) 82:1166–9. doi: 10.56093/ijans.v82i10.24290

[26] Cimpeanu R Castrejon-Pita AA Lim LN Vatish M Georgiou EX. A new flow-based design for double-lumen needles. arXiv. (2023) 160:111832. doi: 10.1016/j.jbiomech.2023.111832, PMID: 37837837

[27] Myers A Kasimanickam R. Laparoscopic artificial insemination in sheep: review and cost benefit analysis. Clin Therio. (2025) 17:11080. doi: 10.58292/CT.v17.11080

[28] Hirsch L Gibney M Berube J Manocchio J. Impact of a modified needle tip geometry on penetration force as well as acceptability, preference, and perceived pain in subjects with diabetes. J Diabetes Sci Technol. (2012) 6:328–35. doi: 10.1177/193229681200600216 22538142 PMC3380774

[29] Dau M Buttchereit I Ganz C Frerich B Anisimova EN Daubländer M. Influence of needle bevel design on injection pain and needle deformation in dental local infiltration anaesthesia—a randomized clinical trial. Int J Oral Maxillofac Surg. (2017) 46:1484–89. doi: 10.1016/j.ijom.2017.06.013 28711309

[30] Tirpan MB Tekin K Cil B Alemdar H Inanc ME Olgac KT. The effects of different PMSG doses on estrus behavior and pregnancy rate in angora goats. Animal. (2019) 13:564–9. doi: 10.1017/S1751731118001908, PMID: 30049294

[31] Andersson M Taponen J Koskinen E Dahlbom M. Effect of insemination with doses of 2 or 15 million frozen-thawed spermatozoa and semen deposition site on pregnancy rate in dairy cows. Theriogenology. (2004) 61:1583–8. doi: 10.1016/j.theriogenology.2003.09.006, PMID: 15036987

[32] Graves WM Dowlen HH Kiess A. Evaluation of uterine body and bilateral uterine horn insemination techniques. J Dairy Sci. (1991) 74:3454–6. doi: 10.3168/jds.S0022-0302(91)78535-4, PMID: 1744275

[33] Perkins NR Hill JR Pedrana RG. Laparoscopic insemination of frozen-thawed semen into one or both uterine horns without regard to ovulation site in synchronized merino ewes. Theriogenology. (1996) 46:541–5. doi: 10.1016/0093-691X(96)00175-6, PMID: 16727921

[34] Anakkul N Suwimonteerabutr J Tharasanit T Khunmanee S Diloksumpan P Berg KD. Sperm distribution and fertilization after unilateral and bilateral laparoscopic artificial insemination with frozen-thawed goat semen. Theriogenology. (2014) 82:1137–44. doi: 10.1016/j.theriogenology.2014.07.032, PMID: 25175152

[35] Kang TC Shen PC Hsing HL Fan GJ Chuang PH Chu FH. The effects of transcervical and laparoscopic artificial insemination using frozen-thawed semen on pregnancy rate, delivery rate and the average litter size in goats. Taiwan Livest Res. (2015) 48:119–24. doi: 10.6991/JTLR

[36] Heinemann L Nguyen T Bailey TS Hassoun A Kulzer B Oliveria T. Needle technology for insulin administration: a century of innovation. J Diabetes Sci Technol. (2023) 17:449–57. doi: 10.1177/19322968211059564, PMID: 34889142 PMC10012366

[37] Præstmark KA Jensen ML Madsen NB Kildegaard J Stallknecht BM. Pen needle design influences ease of insertion, pain, and skin trauma in subjects with type 2 diabetes. BMJ Open Diabetes Res Care. (2016) 4:e000266. doi: 10.1136/bmjdrc-2016-000266, PMID: 28074137 PMC5174793

[38] Aronson R. The role of comfort and discomfort in insulin therapy. Diabetes Technol Ther. (2012) 14:741–7. doi: 10.1089/dia.2012.0038, PMID: 22537418 PMC3409452

