# Supplementary material for: Efficiency of laparoscopic artificial insemination in goats: impact of laparoscopic insemination sheath needle design
Source: Front Vet Sci. 2025 Jun 27;12:1579540. doi: 10.3389/fvets.2025.1579540 (PMC12247530; doi:10.3389/fvets.2025.1579540)
Supplement: Supplementary file 1 [file Data_Sheet_1.pdf]

Supplementary

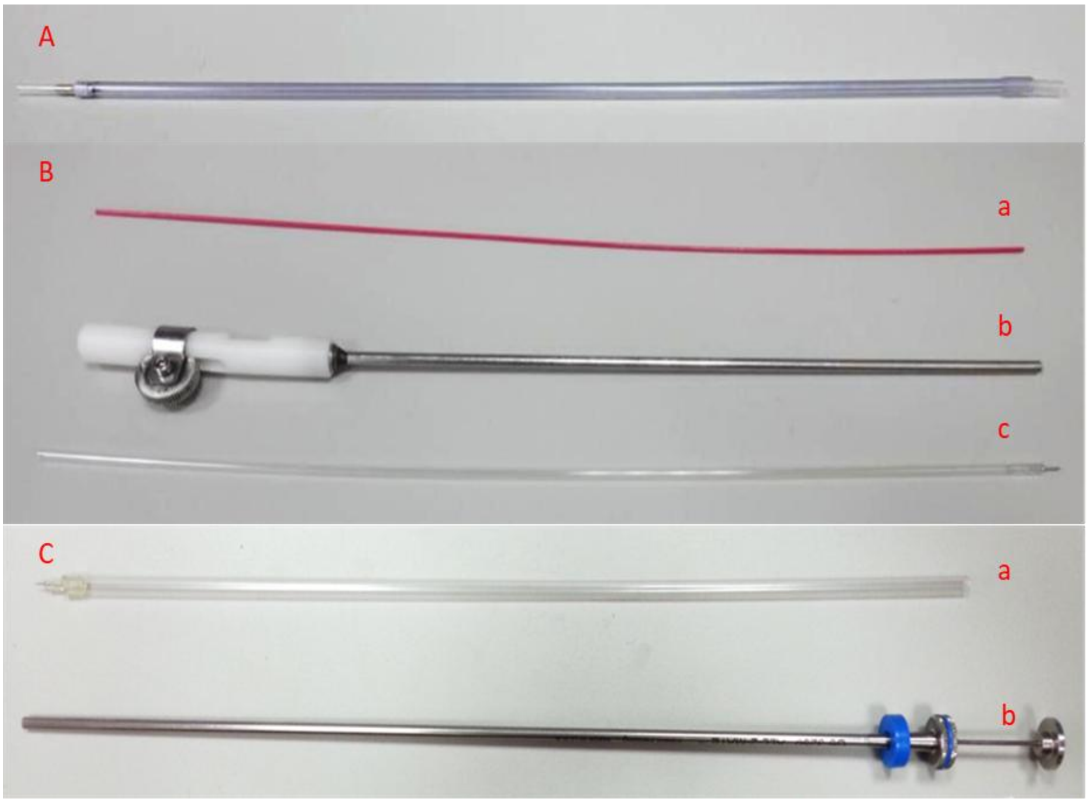

Figure 1. Laparoscopic insemination tubes. (A) Tube manufactured by Minitub, (B) tube manufactured by IMV (a: semen putter, b: injection gun, c: insemination tube), and (C) TLRI tube (a: insemination tube, b: insemination gun).

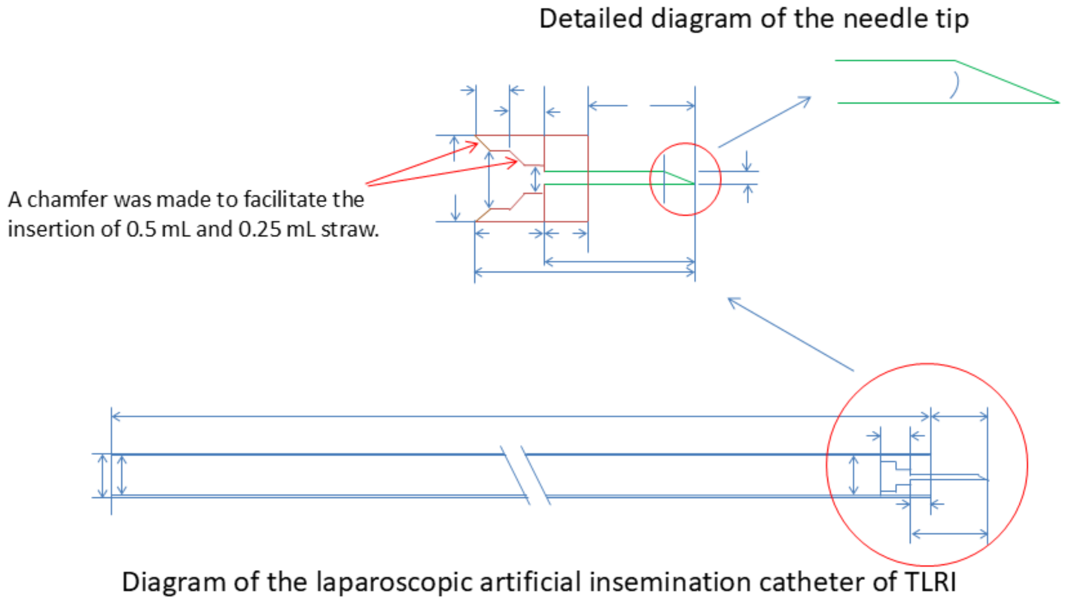

Figure 2. Diagram of the laparoscopic artificial insemination catheter of TLRI. However, due to the involvement of intellectual property rights, it is permissible to omit all detailed specifications.
